# 10,13-Dimethyl-16-oxo-4,5,6,7,8,9,10,11,12,13,14,15,16,17-tetra­deca­hydro-1*H*-cyclo­penta­[*a*]phenanthren-17-yl acetate

**DOI:** 10.1107/S1600536810028412

**Published:** 2010-07-24

**Authors:** Rui Shi, Chun-Sheng Zhang, Rong Huang, Kun Wei

**Affiliations:** a Key Laboratory of Forest Resources Conservation and Use in the Southwest Mountains of China (Ministry of Education), Southwest Forestry University, Kunming 650224, People’s Republic of China; b Zhejiang Apeloa Kangyu Pharmaceutical Co Ltd, Jinhua 322118, People’s Republic of China; c School of Chemical Science and Technology, Key Laboratory of Medicinal Chemistry for Natural Resources (Ministry of Education), Yunnan University, Kunming 650091, People’s Republic of China

## Abstract

In the title compound, C_21_H_30_O_3_, the five-membered ring adopts an envelope conformation, the cyclo­hexene ring displays a half-chair conformation and the two cyclo­hexane rings have normal chair conformations. In the crystal structure, weak inter­molecular C—H⋯O hydrogen bonding links the mol­ecules into supra­molecular chains running along [100].

## Related literature

Rocuronium is a non-depolarizing neuromuscular blocking agent. The title compound was obtained as an inter­mediate during our ongoing investigation of the synthesis of rocuronium bromide; for further information on rocuronium bromide, see: Tuba *et al.* (2002 ▶); Auer (2007 ▶). For the synthesis, see: Tuba (1980 ▶); Newaz & Tcholakian (1984 ▶).
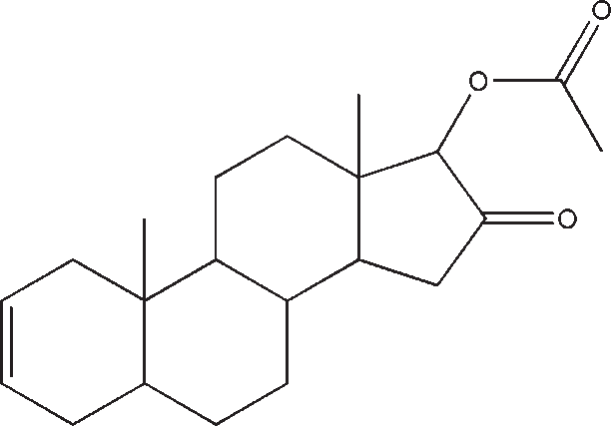


## Experimental

### 

#### Crystal data

C_21_H_30_O_3_
*M* *_r_* = 330.45
Monoclinic, 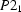

*a* = 7.383 (5) Å
*b* = 13.200 (9) Å
*c* = 9.843 (7) Å
β = 95.687 (10)°
*V* = 954.5 (11) Å^3^
*Z* = 2
Mo *K*α radiation
μ = 0.08 mm^−1^
*T* = 293 K
0.45 × 0.40 × 0.32 mm

#### Data collection

Bruker SMART CCD area-detector diffractometer
5527 measured reflections
1794 independent reflections
864 reflections with *I* > 2σ(*I*)
*R*_int_ = 0.102

#### Refinement

*R*[*F* ^2^ > 2σ(*F* ^2^)] = 0.053
*wR*(*F* ^2^) = 0.136
*S* = 0.86
1794 reflections
221 parameters
1 restraint
H-atom parameters constrained
Δρ_max_ = 0.13 e Å^−3^
Δρ_min_ = −0.11 e Å^−3^

### 

Data collection: *SMART* (Bruker, 2004 ▶); cell refinement: *SAINT* (Bruker, 2004 ▶); data reduction: *SAINT*; program(s) used to solve structure: *SHELXS97* (Sheldrick, 2008 ▶); program(s) used to refine structure: *SHELXL97* (Sheldrick, 2008 ▶); molecular graphics: *SHELXTL* (Sheldrick, 2008 ▶); software used to prepare material for publication: *SHELXTL*.

## Supplementary Material

Crystal structure: contains datablocks I, global. DOI: 10.1107/S1600536810028412/xu2791sup1.cif

Structure factors: contains datablocks I. DOI: 10.1107/S1600536810028412/xu2791Isup2.hkl

Additional supplementary materials:  crystallographic information; 3D view; checkCIF report

## Figures and Tables

**Table 1:** Hydrogen-bond geometry (Å, °)

*D*—H⋯*A*	*D*—H	H⋯*A*	*D*⋯*A*	*D*—H⋯*A*
C19—H19*C*⋯O1^i^	0.96	2.55	3.496 (11)	170

Symmetry code: (i) 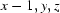.

**Fig. 1.**
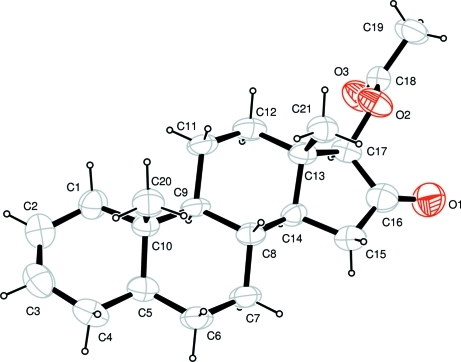
The molecular structure of the title compound, showing the atom labelling scheme and 30% probability displacement ellipsoids.

## supplementary crystallographic information

### Comment

Rocuronium is a nondepolarizing neuromuscular blocking agent, which produces neuromuscular blockade by competing with acetylcholine for cholinergic receptors at the motor end plate (Tuba *et al.*, 2002; Auer, 2007). The title compound was obtained as an intermediate during our ongoing investigation of the synthese of rocuronium bromide. In this paper, we report the crystal structure of the title compound.

The molecular structure of the title compound is shown in Fig. 1. In the molecular structure, the cyclohexene ring displays a half-chair conformtion, the five ring adopts an envelope conformation, and two cyclohexane rings have the normal chair conformation. In the crystal structure weak intermolecular C—H···O hydrogen bonding links the molecules to form the supra-molecular chain running along the [1 0 0] direction (Table 1).

### Experimental

The title compound was synthesized according to the procedure reported by Tuba *et al.* (1980) and by Newaz & Tcholakian (1984). Single crystals were obtained from a mixture of ethyl acetate and petroleum ether by slow evaporation at room temperature.

### Refinement

All H atoms were placed in calculated positions, with C—H = 0.93–0.98 Å, and included in the final cycles of refinement using a riding model, with *U*_iso_(H) = 1.2 (1.5 for methyl groups) times *U*_eq_(C). Friedel paris were merged as no significant anomalous scattering.

### Crystal data

**Table:**

C_21_H_30_O_3_	*F*(000) = 360
*M**_r_* = 330.45	*D*_x_ = 1.150 Mg m^−^^3^
Monoclinic, *P*2_1_	Mo *K*α radiation, λ = 0.71073 Å
Hall symbol: P 2yb	Cell parameters from 864 reflections
*a* = 7.383 (5) Å	θ = 5.7–25.7°
*b* = 13.200 (9) Å	µ = 0.08 mm^−^^1^
*c* = 9.843 (7) Å	*T* = 293 K
β = 95.687 (10)°	Block, colorless
*V* = 954.5 (11) Å^3^	0.45 × 0.40 × 0.32 mm
*Z* = 2	

### Data collection

**Table:**

Bruker SMART CCD area-detector diffractometer	864 reflections with *I* > 2σ(*I*)
Radiation source: fine-focus sealed tube	*R*_int_ = 0.102
graphite	θ_max_ = 25.2°, θ_min_ = 2.1°
φ and ω scans	*h* = −8→8
5527 measured reflections	*k* = −9→15
1794 independent reflections	*l* = −11→11

### Refinement

**Table:**

Refinement on *F*^2^	Secondary atom site location: difference Fourier map
Least-squares matrix: full	Hydrogen site location: inferred from neighbouring sites
*R*[*F*^2^ > 2σ(*F*^2^)] = 0.053	H-atom parameters constrained
*wR*(*F*^2^) = 0.136	*w* = 1/[σ^2^(*F*_o_^2^) + (0.0539*P*)^2^] where *P* = (*F*_o_^2^ + 2*F*_c_^2^)/3
*S* = 0.86	(Δ/σ)_max_ < 0.001
1794 reflections	Δρ_max_ = 0.13 e Å^−^^3^
221 parameters	Δρ_min_ = −0.11 e Å^−^^3^
1 restraint	Extinction correction: *SHELXL97* (Sheldrick, 2008), Fc^*^=kFc[1+0.001xFc^2^λ^3^/sin(2θ)]^-1/4^
Primary atom site location: structure-invariant direct methods	Extinction coefficient: 0.018 (3)

### Special details

**Table:**

Geometry. All esds (except the esd in the dihedral angle between two l.s. planes) are estimated using the full covariance matrix. The cell esds are taken into account individually in the estimation of esds in distances, angles and torsion angles; correlations between esds in cell parameters are only used when they are defined by crystal symmetry. An approximate (isotropic) treatment of cell esds is used for estimating esds involving l.s. planes.
Refinement. Refinement of *F*^2^ against ALL reflections. The weighted *R*-factor *wR* and goodness of fit *S* are based on *F*^2^, conventional *R*-factors *R* are based on *F*, with *F* set to zero for negative *F*^2^. The threshold expression of *F*^2^ > σ(*F*^2^) is used only for calculating *R*-factors(gt) *etc*. and is not relevant to the choice of reflections for refinement. *R*-factors based on *F*^2^ are statistically about twice as large as those based on *F*, and *R*- factors based on ALL data will be even larger.

### Fractional atomic coordinates and isotropic or equivalent isotropic displacement parameters (Å^2^)

**Table:**

	*x*	*y*	*z*	*U*_iso_*/*U*_eq_	
C1	0.2353 (9)	0.8413 (7)	0.3971 (7)	0.089 (2)	
H1A	0.1047	0.8316	0.3809	0.107*	
H1B	0.2756	0.8100	0.4841	0.107*	
C2	0.2742 (13)	0.9525 (7)	0.4068 (8)	0.104 (3)	
H2	0.1975	0.9938	0.4519	0.125*	
C3	0.4149 (14)	0.9942 (7)	0.3531 (9)	0.109 (3)	
H3	0.4306	1.0640	0.3597	0.130*	
C4	0.5469 (11)	0.9345 (7)	0.2835 (8)	0.100 (2)	
H4A	0.6690	0.9545	0.3187	0.120*	
H4B	0.5318	0.9506	0.1868	0.120*	
C5	0.5277 (9)	0.8189 (6)	0.3009 (7)	0.084 (2)	
H5	0.5753	0.8028	0.3949	0.100*	
C6	0.6463 (9)	0.7619 (6)	0.2068 (8)	0.090 (2)	
H6A	0.6053	0.7772	0.1123	0.108*	
H6B	0.7716	0.7842	0.2245	0.108*	
C7	0.6356 (8)	0.6477 (6)	0.2303 (7)	0.087 (2)	
H7A	0.7052	0.6129	0.1660	0.104*	
H7B	0.6896	0.6319	0.3217	0.104*	
C8	0.4396 (8)	0.6097 (5)	0.2132 (6)	0.0669 (18)	
H8	0.3895	0.6192	0.1181	0.080*	
C9	0.3222 (8)	0.6712 (6)	0.3088 (6)	0.0703 (19)	
H9	0.3770	0.6599	0.4023	0.084*	
C10	0.3266 (8)	0.7864 (5)	0.2843 (6)	0.0674 (18)	
C11	0.1274 (8)	0.6266 (6)	0.3010 (7)	0.083 (2)	
H11A	0.0603	0.6610	0.3673	0.100*	
H11B	0.0660	0.6400	0.2111	0.100*	
C12	0.1231 (9)	0.5120 (7)	0.3282 (7)	0.089 (2)	
H12A	0.1719	0.4987	0.4217	0.107*	
H12B	−0.0019	0.4884	0.3170	0.107*	
C13	0.2347 (8)	0.4539 (5)	0.2304 (6)	0.0716 (19)	
C14	0.4277 (8)	0.4979 (5)	0.2504 (6)	0.067 (2)	
H14	0.4674	0.4928	0.3481	0.081*	
C15	0.5425 (9)	0.4225 (6)	0.1788 (7)	0.091 (2)	
H15A	0.5404	0.4378	0.0823	0.110*	
H15B	0.6677	0.4223	0.2195	0.110*	
C16	0.4499 (12)	0.3221 (8)	0.2014 (8)	0.100 (3)	
C17	0.2753 (9)	0.3439 (6)	0.2690 (7)	0.0768 (19)	
H17	0.3009	0.3385	0.3683	0.092*	
C18	0.0718 (10)	0.2130 (6)	0.3117 (10)	0.084 (2)	
C19	−0.0681 (10)	0.1408 (6)	0.2474 (8)	0.116 (3)	
H19A	−0.0850	0.0864	0.3097	0.174*	
H19B	−0.0276	0.1138	0.1650	0.174*	
H19C	−0.1813	0.1758	0.2262	0.174*	
C20	0.2325 (8)	0.8158 (5)	0.1428 (6)	0.083 (2)	
H20A	0.1055	0.7990	0.1384	0.125*	
H20B	0.2876	0.7794	0.0732	0.125*	
H20C	0.2457	0.8873	0.1288	0.125*	
C21	0.1481 (8)	0.4600 (5)	0.0849 (6)	0.083 (2)	
H21A	0.2156	0.4187	0.0273	0.125*	
H21B	0.1489	0.5290	0.0542	0.125*	
H21C	0.0249	0.4361	0.0806	0.125*	
O1	0.4948 (9)	0.2388 (5)	0.1678 (7)	0.129 (2)	
O2	0.1340 (7)	0.2744 (4)	0.2230 (5)	0.0994 (16)	
O3	0.1307 (8)	0.2124 (5)	0.4295 (6)	0.127 (2)	

### Atomic displacement parameters (Å^2^)

**Table:**

	*U*^11^	*U*^22^	*U*^33^	*U*^12^	*U*^13^	*U*^23^
C1	0.089 (5)	0.107 (7)	0.075 (5)	0.001 (5)	0.020 (4)	−0.015 (5)
C2	0.122 (7)	0.091 (7)	0.098 (6)	0.004 (5)	0.007 (5)	−0.003 (5)
C3	0.123 (7)	0.094 (7)	0.104 (7)	−0.012 (7)	−0.012 (6)	−0.010 (6)
C4	0.092 (6)	0.108 (7)	0.099 (6)	−0.036 (5)	0.000 (5)	0.003 (5)
C5	0.067 (5)	0.100 (7)	0.083 (5)	−0.012 (4)	0.000 (4)	0.001 (4)
C6	0.057 (4)	0.096 (6)	0.119 (6)	−0.014 (4)	0.016 (4)	0.008 (5)
C7	0.051 (4)	0.103 (6)	0.109 (6)	−0.010 (4)	0.016 (4)	0.003 (5)
C8	0.052 (4)	0.082 (6)	0.064 (4)	−0.001 (4)	−0.005 (3)	0.015 (4)
C9	0.041 (4)	0.102 (6)	0.069 (5)	−0.014 (4)	0.015 (3)	−0.002 (4)
C10	0.057 (4)	0.083 (6)	0.062 (4)	−0.005 (4)	0.001 (4)	−0.001 (4)
C11	0.047 (4)	0.114 (7)	0.091 (6)	−0.010 (4)	0.020 (4)	−0.005 (5)
C12	0.064 (5)	0.125 (7)	0.079 (5)	−0.025 (4)	0.016 (4)	0.011 (5)
C13	0.057 (4)	0.104 (6)	0.054 (4)	−0.014 (4)	0.005 (3)	−0.002 (4)
C14	0.056 (4)	0.090 (6)	0.058 (4)	−0.001 (4)	0.015 (3)	−0.005 (4)
C15	0.067 (5)	0.102 (6)	0.104 (6)	−0.006 (5)	0.005 (4)	0.009 (5)
C16	0.088 (6)	0.109 (8)	0.099 (6)	0.004 (6)	−0.010 (5)	0.012 (7)
C17	0.068 (5)	0.083 (6)	0.077 (5)	−0.018 (5)	−0.003 (4)	0.014 (4)
C18	0.076 (5)	0.083 (6)	0.094 (6)	−0.002 (5)	0.014 (5)	−0.020 (6)
C19	0.088 (6)	0.096 (6)	0.160 (8)	−0.033 (5)	−0.011 (5)	0.000 (5)
C20	0.076 (4)	0.095 (6)	0.077 (5)	0.000 (4)	−0.003 (4)	0.006 (4)
C21	0.075 (4)	0.104 (6)	0.069 (5)	−0.007 (4)	0.001 (4)	0.002 (4)
O1	0.124 (5)	0.096 (5)	0.168 (6)	0.011 (4)	0.022 (4)	0.013 (4)
O2	0.107 (4)	0.100 (4)	0.087 (4)	−0.037 (3)	−0.008 (3)	0.020 (3)
O3	0.141 (5)	0.142 (5)	0.100 (4)	−0.057 (4)	0.027 (4)	0.001 (4)

### Geometric parameters (Å, °)

**Table:**

C1—C2	1.497 (11)	C11—H11B	0.9700
C1—C10	1.536 (8)	C12—C13	1.534 (8)
C1—H1A	0.9700	C12—H12A	0.9700
C1—H1B	0.9700	C12—H12B	0.9700
C2—C3	1.331 (10)	C13—C21	1.512 (8)
C2—H2	0.9300	C13—C17	1.522 (9)
C3—C4	1.473 (10)	C13—C14	1.534 (8)
C3—H3	0.9300	C14—C15	1.524 (9)
C4—C5	1.544 (10)	C14—H14	0.9800
C4—H4A	0.9700	C15—C16	1.517 (11)
C4—H4B	0.9700	C15—H15A	0.9700
C5—C6	1.533 (8)	C15—H15B	0.9700
C5—C10	1.538 (8)	C16—O1	1.204 (9)
C5—H5	0.9800	C16—C17	1.536 (10)
C6—C7	1.528 (9)	C17—O2	1.429 (7)
C6—H6A	0.9700	C17—H17	0.9800
C6—H6B	0.9700	C18—O3	1.197 (8)
C7—C8	1.525 (8)	C18—O2	1.306 (9)
C7—H7A	0.9700	C18—C19	1.500 (9)
C7—H7B	0.9700	C19—H19A	0.9600
C8—C14	1.525 (8)	C19—H19B	0.9600
C8—C9	1.568 (8)	C19—H19C	0.9600
C8—H8	0.9800	C20—H20A	0.9600
C9—C10	1.541 (8)	C20—H20B	0.9600
C9—C11	1.549 (7)	C20—H20C	0.9600
C9—H9	0.9800	C21—H21A	0.9600
C10—C20	1.543 (8)	C21—H21B	0.9600
C11—C12	1.536 (9)	C21—H21C	0.9600
C11—H11A	0.9700		
			
C2—C1—C10	114.4 (7)	C9—C11—H11B	108.8
C2—C1—H1A	108.7	H11A—C11—H11B	107.7
C10—C1—H1A	108.7	C13—C12—C11	111.2 (5)
C2—C1—H1B	108.7	C13—C12—H12A	109.4
C10—C1—H1B	108.7	C11—C12—H12A	109.4
H1A—C1—H1B	107.6	C13—C12—H12B	109.4
C3—C2—C1	122.2 (8)	C11—C12—H12B	109.4
C3—C2—H2	118.9	H12A—C12—H12B	108.0
C1—C2—H2	118.9	C21—C13—C17	110.0 (5)
C2—C3—C4	122.8 (8)	C21—C13—C12	111.4 (5)
C2—C3—H3	118.6	C17—C13—C12	115.2 (6)
C4—C3—H3	118.6	C21—C13—C14	113.6 (5)
C3—C4—C5	113.9 (7)	C17—C13—C14	99.9 (5)
C3—C4—H4A	108.8	C12—C13—C14	106.3 (5)
C5—C4—H4A	108.8	C15—C14—C8	118.2 (5)
C3—C4—H4B	108.8	C15—C14—C13	104.2 (5)
C5—C4—H4B	108.8	C8—C14—C13	114.3 (6)
H4A—C4—H4B	107.7	C15—C14—H14	106.4
C6—C5—C10	113.7 (6)	C8—C14—H14	106.4
C6—C5—C4	110.7 (6)	C13—C14—H14	106.4
C10—C5—C4	111.2 (6)	C16—C15—C14	102.9 (6)
C6—C5—H5	106.9	C16—C15—H15A	111.2
C10—C5—H5	106.9	C14—C15—H15A	111.2
C4—C5—H5	106.9	C16—C15—H15B	111.2
C7—C6—C5	110.6 (6)	C14—C15—H15B	111.2
C7—C6—H6A	109.5	H15A—C15—H15B	109.1
C5—C6—H6A	109.5	O1—C16—C15	128.1 (8)
C7—C6—H6B	109.5	O1—C16—C17	123.8 (9)
C5—C6—H6B	109.5	C15—C16—C17	108.1 (8)
H6A—C6—H6B	108.1	O2—C17—C13	114.5 (5)
C8—C7—C6	111.8 (6)	O2—C17—C16	111.1 (6)
C8—C7—H7A	109.3	C13—C17—C16	102.9 (6)
C6—C7—H7A	109.3	O2—C17—H17	109.4
C8—C7—H7B	109.3	C13—C17—H17	109.4
C6—C7—H7B	109.3	C16—C17—H17	109.4
H7A—C7—H7B	107.9	O3—C18—O2	122.2 (8)
C14—C8—C7	111.6 (5)	O3—C18—C19	125.0 (9)
C14—C8—C9	108.0 (5)	O2—C18—C19	112.7 (8)
C7—C8—C9	109.8 (5)	C18—C19—H19A	109.5
C14—C8—H8	109.1	C18—C19—H19B	109.5
C7—C8—H8	109.1	H19A—C19—H19B	109.5
C9—C8—H8	109.1	C18—C19—H19C	109.5
C10—C9—C11	113.7 (6)	H19A—C19—H19C	109.5
C10—C9—C8	113.3 (5)	H19B—C19—H19C	109.5
C11—C9—C8	109.9 (5)	C10—C20—H20A	109.5
C10—C9—H9	106.5	C10—C20—H20B	109.5
C11—C9—H9	106.5	H20A—C20—H20B	109.5
C8—C9—H9	106.5	C10—C20—H20C	109.5
C1—C10—C5	106.3 (6)	H20A—C20—H20C	109.5
C1—C10—C9	109.5 (6)	H20B—C20—H20C	109.5
C5—C10—C9	107.1 (5)	C13—C21—H21A	109.5
C1—C10—C20	110.1 (5)	C13—C21—H21B	109.5
C5—C10—C20	111.7 (5)	H21A—C21—H21B	109.5
C9—C10—C20	112.0 (5)	C13—C21—H21C	109.5
C12—C11—C9	113.6 (6)	H21A—C21—H21C	109.5
C12—C11—H11A	108.8	H21B—C21—H21C	109.5
C9—C11—H11A	108.8	C18—O2—C17	118.7 (6)
C12—C11—H11B	108.8		
			
C10—C1—C2—C3	−19.1 (10)	C11—C12—C13—C21	−67.3 (7)
C1—C2—C3—C4	−1.9 (13)	C11—C12—C13—C17	166.6 (5)
C2—C3—C4—C5	−10.3 (11)	C11—C12—C13—C14	57.0 (7)
C3—C4—C5—C6	170.4 (6)	C7—C8—C14—C15	−54.7 (8)
C3—C4—C5—C10	43.0 (8)	C9—C8—C14—C15	−175.5 (5)
C10—C5—C6—C7	−57.3 (8)	C7—C8—C14—C13	−177.9 (5)
C4—C5—C6—C7	176.7 (6)	C9—C8—C14—C13	61.3 (6)
C5—C6—C7—C8	55.4 (8)	C21—C13—C14—C15	−70.3 (7)
C6—C7—C8—C14	−174.4 (6)	C17—C13—C14—C15	46.7 (6)
C6—C7—C8—C9	−54.7 (7)	C12—C13—C14—C15	166.8 (5)
C14—C8—C9—C10	178.5 (5)	C21—C13—C14—C8	60.2 (7)
C7—C8—C9—C10	56.5 (7)	C17—C13—C14—C8	177.3 (5)
C14—C8—C9—C11	−53.2 (7)	C12—C13—C14—C8	−62.7 (7)
C7—C8—C9—C11	−175.1 (6)	C8—C14—C15—C16	−161.0 (5)
C2—C1—C10—C5	49.1 (8)	C13—C14—C15—C16	−32.9 (7)
C2—C1—C10—C9	164.4 (6)	C14—C15—C16—O1	−177.1 (9)
C2—C1—C10—C20	−72.0 (7)	C14—C15—C16—C17	6.5 (7)
C6—C5—C10—C1	173.3 (7)	C21—C13—C17—O2	−42.2 (7)
C4—C5—C10—C1	−60.9 (7)	C12—C13—C17—O2	84.6 (6)
C6—C5—C10—C9	56.3 (7)	C14—C13—C17—O2	−162.0 (5)
C4—C5—C10—C9	−177.9 (5)	C21—C13—C17—C16	78.5 (6)
C6—C5—C10—C20	−66.6 (8)	C12—C13—C17—C16	−154.6 (6)
C4—C5—C10—C20	59.2 (8)	C14—C13—C17—C16	−41.3 (6)
C11—C9—C10—C1	63.0 (6)	O1—C16—C17—O2	−31.4 (11)
C8—C9—C10—C1	−170.6 (5)	C15—C16—C17—O2	145.2 (6)
C11—C9—C10—C5	177.9 (5)	O1—C16—C17—C13	−154.4 (8)
C8—C9—C10—C5	−55.7 (6)	C15—C16—C17—C13	22.2 (7)
C11—C9—C10—C20	−59.4 (7)	O3—C18—O2—C17	−0.9 (11)
C8—C9—C10—C20	67.0 (6)	C19—C18—O2—C17	−176.8 (6)
C10—C9—C11—C12	−178.9 (6)	C13—C17—O2—C18	−127.4 (6)
C8—C9—C11—C12	53.0 (7)	C16—C17—O2—C18	116.5 (7)
C9—C11—C12—C13	−56.0 (7)		

### Hydrogen-bond geometry (Å, °)

**Table:**

*D*—H···*A*	*D*—H	H···*A*	*D*···*A*	*D*—H···*A*
C19—H19C···O1^i^	0.96	2.55	3.496 (11)	170

Symmetry codes: (i) *x*−1, *y*, *z*.
